# What if the upwelling weakens? Effects of rising temperature and nutrient depletion on coastal assemblages

**DOI:** 10.1007/s00442-024-05571-6

**Published:** 2024-06-05

**Authors:** Axel Chabrerie, Francisco Arenas

**Affiliations:** Interdisciplinary Centre of Marine and Environmental Research (CIIMAR), University of Porto, Matosinhos, Portugal

**Keywords:** Ecosystem functioning, Primary productivity, Climate change, Warming, Macroalgae

## Abstract

Surface temperature of the oceans has increased globally over the past decades. In coastal areas influenced by eastern boundary upwelling systems (EBUS), winds push seawater offshore and deep, cold and nutrient-rich seawater rise towards the surface, partially buffering global warming. On the North coast of Portugal, the NW Iberian upwelling system allows extensive kelp forests to thrive in these “boreal-like” conditions, fostering highly diverse and productive communities. However, the warming of the upper layer of the ocean may weaken this upwelling, leading to higher sea surface temperature and lower nutrient input in the coastal areas. The effects of these changes on the structure and function of coastal ecosystems remain unexplored. The present study aimed to examine the combined effects of elevated temperature and nutrient depletion on semi-naturally structured assemblages. The eco-physiological responses investigated included growth, chlorophyll fluorescence and metabolic rates at the levels of individual species and whole assemblages. Our findings showed interactive effects of the combination of elevated temperature with nutrient depletion on the large canopy-forming species (i.e., kelp). As main contributor to community response, those effects drove the whole assemblage responses to significant losses in productivity levels. We also found an additive effect of elevated temperature and reduced nutrients on sub-canopy species (i.e., *Chondrus crispus*), while turfs were only affected by temperature. Our results suggest that under weakening upwelling scenarios, the ability of the macroalgal assemblages to maintain high productivity rates could be seriously affected and predict a shift in community composition with the loss of marine forests.

**Supplementary Information:**

The online version contains supplementary material available at 10.1007/s00442-024-05571-6.

## Introduction

Rocky coastal habitats support extensive and diverse seaweed communities with high ecological relevance known as marine forests (Wernberg and Filbee-Dexter 2019). These communities are characterized by large foundation species such as kelps or other seaweeds with similar functional properties, e.g., sub-tidal large dominant canopy-forming species with high primary productivity of the genus *Cystoseira*, *Fucus*, *Saccorhiza* and *Sargassum* among others (Dayton 1975; Jones et al. 1994; Coleman and Wernberg 2017). Marine forests are highly complex 3-dimensional submerged canopies which provide refuge for multiple species of mammals, crustaceans, fishes, other seaweeds and epibiota, together founding highly diverse assemblages (Mann 1973; Steneck et al. 2002). In addition, these communities have important socio-economical value as they create fishing grounds that support coastal fishing industries across the world (Smale et al. 2013; Bertocci et al. 2015). Besides these ecosystem-supporting and provisioning services, marine forests play an active role in mitigating the effects of climate change on a broad scale (Krause-Jensen and Duarte 2016). Indeed, marine forests, namely those formed by kelps, are thought to be among the most productive systems on Earth and fundamental in the carbon fixation balance of coastal areas. Marine forests net primary productivity may reach > 3000 g C m^−2^ year^−1^ and the global primary productivity of this biome was projected to be around 1,521 TgC y^−1^(Gao & Mckinley 1994; Steneck et al. 2002). Recently, Duarte et al. (2022) updated the estimation of NPP to 1320 TgC y^−1^. About 44% of this primary productivity is exported, and their relevance in carbon sequestration and blue carbon is now starting to be recognized (Krause-Jensen et al. 2018). Also, marine forest species like other habitat formers may have a positive effect on associated species by ameliorating environmental stress and it is suggested that could be managed as a nature-based solution against climate driven loss of biodiversity (Bulleri et al. 2018).

Marine forests, particularly those characterized by cold temperature species like kelps, are facing rising threats due to climate change (Harley et al. 2012; Smale 2020). Anthropogenic activities are increasing ocean temperatures (about 1 °C since pre-industrial time) and the Intergovernmental Panel on Climate Change is expecting average global warming of sea surface temperatures (SST) between 1.5 and more than 4 °C at the end of the century (IPCC 2021). Facing these increasingly stressful environments, organisms with phenotypes that are more thermal resilient will survive and proliferate. Above certain thresholds, organisms will have to migrate or to become locally extinct if the timing or speed at which stress occurs is not allowing acclimatization and adaptation. (Parmesan 2006). Sessile ectothermic species with cold seawater temperature affinity like kelps are particularly affected by temperature increase which regulates the rate of enzymatic reactions and subsequently metabolic rates (Gerard 1997; Allakhverdiev et al. 2008). For instance, temperature and respiration rates are positively correlated, and photosynthetic capacity is promoted when temperature is moderately increased (Larkum et al. 2003). However, beyond certain thresholds, temperature increases may reduce the maximum rate (V_max_) and substrate affinity (K_m_) of key enzymes such as RuBisCO leading to reduced photosynthetic capacity (Davison 1991). Moreover, supra-optimal temperatures can lead to protein denaturation and impairment of damage protection systems which irreversibly affect the integrity of the photosynthetic apparatus (Takahashi and Murata 2008). The metabolic scaling theory predicts that metabolic rate of species ultimately governs most of the higher ecological patterns such has reproduction and distribution (Brown et al. 2004; Kordas et al. 2011). Those adverse effects on key metabolic process such as photosynthesis, ultimately result in reduced growth and overall fitness which in turn affect seaweed population dynamic (Kübler and Davison 1995; Biskup et al. 2014). Furthermore, these adverse effects are likely undermining the resilience of seaweed ecosystems, rendering them more susceptible to additional stressors, such as herbivory pressure or extreme weather events (Dayton et al. 1999; Smale 2020). Consequently, over the past few decades, there has been a global decline in kelp forests, accompanied by a gradual displacement with ecologically less-complex turf-dominated assemblages. This shift has led to diminished habitat quality, reduced carbon fixation, and decreased biodiversity (Filbee-Dexter and Wernberg 2018; Coleman et al. 2020).

Cold and nutrient-rich seawaters resulting from upwelling systems are well suited for kelp species, and variation in the intensity of upwelling modifying thermal variability and nutrient availability are key in the dynamics of kelp populations (Pérez-Matus et al. 2017). Understandably, nutrients are essential for seaweed growth as they are used for the synthesis of a large variety of essential biomolecules including chlorophyll and other photo-pigments (Chapman et al. 1978; Wiencke and Bischof 2013). Typically, the decrease of nutrient content in summer is associated with the onset of senescence in *Laminaria* species (Chapman and Craigie 1977). It is also suggested that greater nutrient availability increases thermal tolerance, suggesting synergistic interactions among those drivers in kelp biology (Gao et al. 2013; Gerard 1997). For instance, in the well-studied southern California upwelling system, the retraction of kelp forests has been associated with intense events of El Niño leading to the warming of surface water and intense stratification of the water column. The combined adverse effects of high temperature and low nutrient availability associated with these extreme events ultimately result in negative growth and consequently local loss of populations (Gagné et al. 1982; Tegner et al. 1996).

In the north of Portugal, the presence of the Northwest (NW) Iberian upwelling system creates a sort of “boreal refuge”, bringing cold and nutrient-rich seawater inshore and stimulating the occurrence of cold temperature affinity species at relative low latitude, including highly productive and diverse kelp assemblages (Tuya et al. 2012). Eastern boundary upwelling systems around the world are facing changes in their intensities, and the NW Iberian upwelling system is predicted to weaken in the near future because of intense surface heating leading to higher stratification (Sydeman et al. 2014; Sousa et al. 2020). This will result not only in seawater warming but also in the reduction in the input of nutrients (Sousa et al. 2020). In the last decades, growing evidence of species distribution shifts in this area has emerged (Lima et al. 2007; Díez et al. 2012; Fernández 2016; Piñeiro-Corbeira et al. 2016; Casado-Amezúa et al. 2019; de Azevedo et al. 2023). Some cold temperature affinity seaweed species are retracting their southern distribution ranges, or are present in reduced densities or confined to small areas where the conditions are more suitable (Viejo et al. 2011; Duarte et al. 2013). Likewise, kelps southern distribution range is retracting as well, and empirical evidences suggest that this phenomenon will continue as the NW Iberian upwelling system is decreasing in intensity leading to warmer water and low nutrient availability (Lima et al. 2007; Fernández 2011; Tuya et al. 2012; Franco et al. 2018; Casado-Amezúa et al. 2019; de Azevedo et al. 2023). Because upwelling dynamics drive the condition of kelp forests in these coastlines, it is critical that we gain a greater understanding of how current and future environmental stressors will affect not only the structure but also the functioning of these coastal assemblages.

In this experiment, we explored the combined effects of ocean warning and nutrient depletion on the structure and functioning of seaweed semi-natural assemblages (i.e., combination of naturally colonized boulders and individual thalli of seaweeds in a mesocosm). The individual and combined effects of such stressors and the consequences on ecosystems are still not well understood and highly variable depending on the local environmental context (Wernberg et al. 2012). We conducted a fully factorial mesocosm experiment to investigate the effects of elevated temperature and nutrient depletion on semi-natural sub-tidal algal communities. These communities consisted of rock pool boulder communities and two canopy-forming species, and allowed us to assess the effects of predicted weakened upwelling on a realistic assemblage of species. Our experimental responses included individual effects, such as growth in biomass of the fronds, photosynthetic performance (using Pulse amplitude modulated (PAM) fluorometer) and primary productivity of individual fronds. In addition, to individual effects, community response was assessed by measuring primary productivity and respiration rates of the whole assemblage. By combining individual- and community-level responses, we aimed to provide insights on how single species performance contributes to community-level responses (Maxwell and Johnson 2000; Tait et al. 2017). We expected that both elevated temperature and nutrient depletion would negatively affect the performance and productivity of the species and assemblages. There is some evidence that nutrient concentration may affect temperature tolerance in *Laminaria* species (Gerard 1997; Gao et al. 2013). Hence, our hypothesis is that both drivers (temperature and nutrients) will have negative and non-additive effects on the structure and functioning of the communities.

## Materials and methods

### Species and assemblages’ collection

Our model communities were assembled using seaweed colonized rock pool boulders and thalli of *Laminaria ochroleuca* and *C. crispus* collected on the same rocky shore. Large boulders were collected from rockpools at Praia Norte (Viana do Castelo, Portugal; 41°41′48ʹʹ N, 08°51′11ʹʹ W), a slate-dominated shore with abundant low-tide channels and rock pools providing access to shallow sub-tidal assemblages. The use of boulders bearing natural assemblages was thought to be more realistic in studying community response to stress and was preferred to fully synthetic assemblages (see Vye et al. 2015; Rodriguez et al. 2016). Indeed, this approach combines the advantages of small mesocosm studies in controlled environments with some of the advantages of field studies which use realistic species assemblages with shared ecological histories (Arenas et al. 2009). Boulders were selected based on their size and species composition in order to keep maximum degree of similarity between them. Each of them holds well-developed assemblages with similar species composition and was selected to have a similar volume (mean ± SE = 1100 ± 2.6 cm^3^, *N* = 20). The composition of the assemblages inhabiting the boulders was dominated by *Corallina* sp. and *Lithophyllum incrustans* both representing on average 58% of the whole assemblage biomass (Supplementary data, Table 1). Species richness in the boulders was on average 5.9 ± 0.35 spp (mean ± SE, *N* = 20). All the boulders were brought to the lab in cool boxes and rinsed in a freshwater bath to remove grazers and sediment.

In addition, 20 young sporophytes of the kelp *L. ochroleuca* were collected by gently scraping the holdfast from its substrate and transported within a cool box to the laboratory where grazers and other epiphytic organisms were removed with freshwater. Individuals were selected with comparable size (mean weight ± SE = 17.71 ± 5.33 g, *N* = 20) and appearance so that none of the samples were fertile (i.e., with sori on their blades). Similarly, 40 thalli of *C. crispus* were collected and transported to the laboratory as above (mean weight ± SE = 1.91 ± 0.69 g, *N* = 40). Using these boulders' natural assemblages and the thalli, we built semi-synthetic assemblages including on each assemblage one young sporophyte of *L. ochroleuca*, two thalli of *C. crispus* and a boulder with its complex turf type assemblage. Thalli of *Laminaria* and *Chondrus* were individually tagged and attached to a mesh (15 mm mesh size) to keep thalli upright at the bottom of the mesocosms and mimic natural assemblages (similar approach in Vye et al. 2015). All the biological assemblages were acclimated at 15 °C natural seawater for seven days prior to the start of the experiment. The experiment was done at the indoor experimental facilities of BOGA-CIIMAR in spring (April to May 2021) and lasted 40 days.

### Experimental set-up

To test for individual and combined effects of nutrient depletion and high temperatures, experimental assemblages were exposed to four different treatments consisting of two temperature levels: 15 °C & 19 °C and two different nutrient concentrations (hereafter, Nut- and Nut +). 15 °C is a rounded annual average seawater temperature in the NW Iberia coastal area, and 19 °C as an average temperature predicted by the IPCC RCP 8.5 scenario “business as usual” (IPCC 2021). Nutrient levels were selected based on the typical values observed in the area, which in some seasons may exhibit considerable variability due to the strong fluctuations in nutrient concentrations driven by the NW Iberian upwelling system. (See details below).

The experimental set-up included 20 mesocosms, with each treatment having 5 replicated assemblages. These mesocosms were polypropylene tanks (60 × 40 × 32 cm) capable of holding 60 L of seawater. Individual turbulent aeration was supplied to each mesocosm to enhance the mixing of seawater and counteract potential pH fluctuations. Each mesocosm was covered with LED-equipped lid that provided a central irradiance level of 65 µE m^−2^ s^−1^. The system was a close seawater circulation system with 500 L head tanks for each treatment for a total of 4 head tanks. Seawater close system was used because of the difficulty to maintain a low nutrient treatment through a fully open seawater. Seawater in the header tanks was filtered in a small mesocosm placed by the head tanks with 5 microns polypropylene microfibers cartridge mechanical filters and with a UV filter to reduce any biological activity before being pumped to the individual mesocosm through small pumps (Syncra 70 l h^−1^, Sicce®). Thus, water was constantly circulating inside mesocosms. Despite the mechanical and biological filtration of the seawater before returning to the mesocosms, we have to acknowledge that treatments were not strictly independent as they shared seawater. At the beginning of the experiment and in order to create the two different nutrient experimental levels, we prepared two sets of artificial water for each treatment by mixing sea-salt (Sea-salt, Aquamedic®) in filtered 1000 L dechlorinated water (i.e., 1000 L of enriched nutrient water and 1000 L of depleted water) using a large 1000 L tanks. Subsequently, the artificial water was transferred into the respective head tanks. Nutrient enriched F2 medium was added every three days (0.05ml_sol_ L^−1^) into the head tanks of Nut + treatment, whereas in Nut- treatment, no nutrients were added. Nitrate and phosphate concentrations were determined by spectrophotometry every two weeks according to the methodology described by Grasshoff et al. (Grasshoff et al. 1999) and nitrate values were determined using an adaptation of the spongy cadmium technique described by Jones (Jones 1984). Nitrate values were in average 25.42 mg L^−1^ (± 5.36, *N* = 10) and phosphate values in average 2.6 (± 0.8, *N* = 10) in the nut + treatment. In the Nut- treatment, nutrient concentration was kept at 1.6 (± 0.42, *N* = 10) for nitrates and 0.032 (± 0.01, *N* = 10) for phosphates. These concentrations were little bit lower than those used by Franco et al (2018) in their experiments with *L. ochroleuca* but are in line with the natural concentration of nutrients in the area of study during summer with and without upwelling conditions (J. N. Franco & F. Arenas per. comm., Brito et al. 2020).

Temperature was controlled at each mesocosms using thermostats (STC-100) connected to seawater coolers (Titan 2000, TH-200, Aquamedic®) coupled to the head tanks and seawater heaters placed in each mesocosm. Temperature was recorded every 15 min using temperature loggers (EnvLogger, Electricblue®). The temperature ranged between 14.9 and 15.1 in the 15 °C treatment and between 18.9 and 19.1 in the 19 °C treatment (mean daily temperatures ± SE = 15.02 ± 0.15; 18.9 ± 0.02, *N* = 40). Additionally, salinity and pH values were assessed every 4 days using a multi-parameter probe (Hach® HQ40) and salinity was adjusted to 35 PSU by adding dechlorinated water to the reservoir tanks when needed.

### Final functional responses

#### Species individual growth

*Laminaria ochroleuca* and *C. crispus* were individually tagged at the beginning of the experiment using numbered plastic tags attached with cable ties. Individual growth was estimated by estimating wet weight (WW) of each individual thallus at the start and at the end of the experiment. WW was determined after removing the excess water by gentle shaking and employing absorbent paper. Growth rates (g WW d^−1^) were calculated as the difference between final and initial wet weight of each thalli divided by the experiment duration in days.

#### Chlorophyll fluorescence

In vivo chlorophyll *a* fluorescence of photosystem II was measured using a portable pulse amplitude modulated fluorometer (Junior-PAM, Walz®). Individuals of *L. ochroleuca* and *C. crispus* were dark-adapted for 15 min. After this period, F_o_ (basal florescence) and F_m_ (maximal florescence) were measured in order to calculate the maximum quantum yield (F_v_/F_m_) of photosystem II, using the following formula (Baker 2008):

$$\frac{Fv}{Fm}=(\frac{Fm}{Fo})/Fm$$

A clip disk was use to insure that the optic fiber was maintained on top of the thalli always in the same position. Two saturating pulses (2000–3000 µE m^−2^ s^−1^) were applied on separate spots on each individual above the meristem on *L.ochroleuca* and in the central area of the blade for *C. crispus*, and the average value was used as an estimate of photosynthetic efficiency of the algae.

#### Primary productivity

At the end of the experiment, we quantified the primary productivity rates of the entire assemblage by measuring oxygen production within incubation chambers at various irradiance levels, i.e., Productivity–Irradiance curves (P–I curves). This enabled us to assess the respiration rates of primary producers, ascertain the maximum productivity of the assemblages, and evaluate their efficiency under conditions of limited light availability. All these metabolic rates were assessed by measuring oxygen production/consumption in chambers with filtered seawater. Incubation chambers consisted of 14 L sealed acrylic chambers partially submersed inside a 140 L bath in which temperature was kept at 15 and 19 °C (± 0.7) using a thermostat and a seawater chiller. Each chamber was equipped with an optical dissolved oxygen probe connected to a data logger (Hach® HQ40) which recorded oxygen concentration every minute. The setting included a light source composed of 8 LED lamps (Cube50 plant, Aquamedic®) placed above the chambers and controlled electronically allowing measurements of oxygen production at six different irradiance levels: 0 (dark), 120, 195, 340, 625, and 900 µE m^−2^ s^−1^. Light intensity inside the incubation chambers was measured using a scalar quantum sensor and a logger (ULM-500 universal light meter, Waltz®). Oxygen concentrations were corrected by the volume of seawater in the container, accounting for the variability in the volume of the boulders and algal assemblages. Respiration and primary productivity rates at each light intensity were estimated using the package LolinR which implement local regression technic for estimation of monotonic rates (C. Olito et al. 2017).

P–I curves from productivity data were fitted using the R package phytotools (Revell 2012) using the model of Eilers and Peeters (1988) following the equation:

$$GPP=\frac{{{P}_{max}}^{2}\left(1+\beta \right)\left(E/{E}_{opt}\right)}{{\left(E/{E}_{opt}\right)}^{2}+2\beta E/{E}_{opt}+1}$$

where *P*_*max*_ is the maximum productivity at selected irradiance and temperature, *E*_*opt*_ the optimum light intensity and *β* the slope of photo-inhibition. We then extracted from the fit three different photosynthesis–irradiance parameters: maximum gross primary productivity (GPP_max_), the maximum gross productivity estimated from the PI curve (µmol O_2_ g^−1^DW min^−1^); community respiration as the oxygen consumption during the dark period (µmol O_2_ g^−1^DW min^−1^) and photosynthetic efficiency at low irradiance (α-alpha) as the slope of the P–I relationship for the light limited portion of the curve (µmolO_2_ g^−1^DW min^−1^ µE^−1^ m^−2^ s^−1^).

In addition to the assemblage level measures, P–I curves were determined for individual components of the assemblages using single thalli and a similar methodology as above. The assemblages were divided in the three most abundant morpho-functional groups: the canopy represented by *L. ochroleuca*, sub-canopy by *C. crispus* and the turf represented mostly by *Corallina* sp. Incubation chambers consisted of 0.06 Land 0.2 L (depending on the size of the thalli) acrylic chambers submersed inside a 40 L temperature-controlled bath. Each chamber was equipped with optical oxygen sensors (OxyPro®, PreSens). We used simultaneously 4 chambers with 4 optical sensors placed inside a light-controlled cabinet able to perform seven different light intensity levels: 0, 20, 150, 440, 610, 900, and 1300 μE m^−2^ s^−1^.

Boulders were scraped at the end of the incubation procedure, and seaweeds were sorted by species. All the biomass samples collected were dried at 60 °C for 48 h to obtain estimation of dry weight (DW) and correct values of P–I curves. Calcium carbonate from calcareous algae like *Corallina *sp. and *L. incrustans* was dissolved in 5% concentrated HCl solution for 48 h, rinsed with freshwater and dried as above.

### Data analysis

Potential differences in the composition of the assemblages across treatments were tested using Permutational Multivariate Analysis of Variance (PERMANOVA; Anderson 2017) on root square transformed biomass and Bray–Curtis similarity distances of the species biomass hold in the mesocosms. For the experimental responses, we conducted a two-way fully factorial ANOVA to investigate the individual and interactive effects of the temperature and nutrient treatments on the growth (gWW d^−1^), maximum quantum yield, GPP_max_, and respiration rates (µmolO_2_ gDW^−1^ min^−1^) and alpha (µmolO_2_ g^−1^DW min^−1^ µE^−1^ m^−2^ s^−1^) of our full assemblages and individual components of those assemblages. Normality was checked by performing Shapiro–Wilk test and assumption of heteroscedasticity in the variance was tested through Cochran’s test. If significant differences were detected between groups, Tukey post hoc test was performed to compare significant mean. All statistical analyses and plots were implemented on R software (R Development Core Team 2020) and the GAD package (Sandrini-Neto and Camargo 2022).

## Results

### Analysis of the structure of the assemblages

Assemblages from four different treatments included in this study did not differ in composition (PERMANOVA, Pseudo-F_3,16_: 0.941, *p* = 0.53). Thus, potential treatment differences were not caused by differences in the structure of the assemblages.

### Growth of canopy and sub-canopy species

**Fig. 1:**
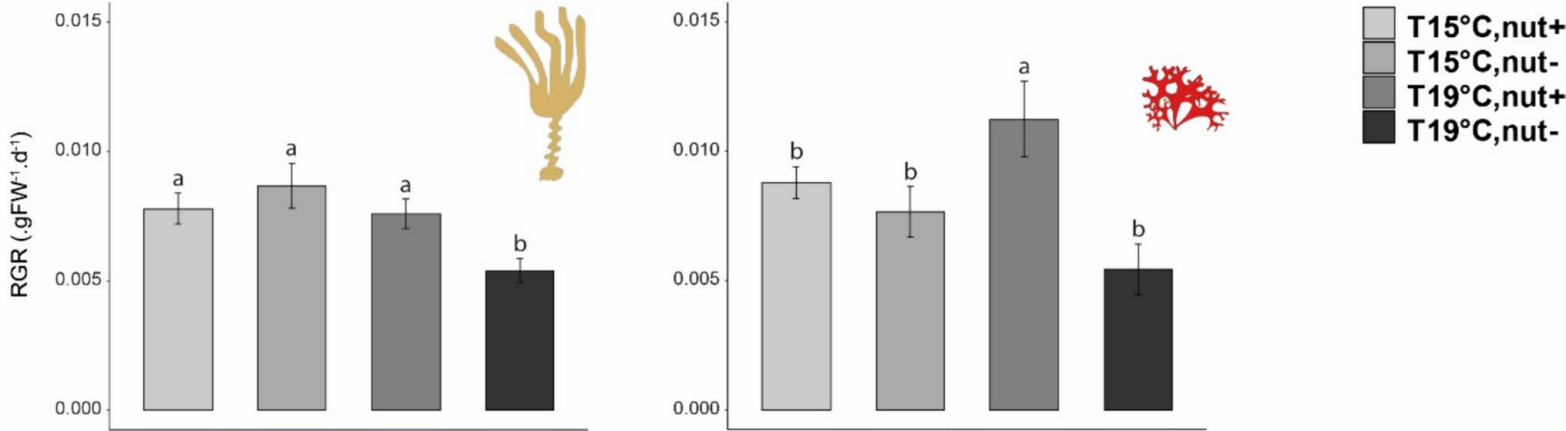
Mean relative growth of *Laminaria ochroleuca* and *Chondrus crispus* calculated over the 4 weeks of experiment. Values are presented as means ± SD (*N* = 5) and expressed in gram of wet weight per day (gWW d^−1^). *p *values from two-way ANOVA (*p* ≤ 0.05). If interaction was significant, Tukey post hoc test was performed to detect differences between groups. Different letters denote for significant differences

The analysis of variance on the relative growth rates of *L. ochroleuca* revealed a significant effect of the interaction Temperature x Nutrients (Fig. 1, Table 1a), suggesting the existence of interactive effects for the two experimental treatments tested. In fact, throughout the course of the experiment, growth of the *L. ochroleuca* at 19 °C and depleted nutrient treatment were significantly lower (mean ± SD = 0.005 ± 0.001 gWW d^−1^, *N* = 5) than the growth rates of all other experimental groups (mean ± SD = 0.008 ± 0.001 gWW d^−1^, *N* = 15). In the case of *C. crispus*, the analysis of variance detected also a significant interaction among treatments, but the nature of this interaction was different (Fig. 1, Table 1b). Hence, when thalli were exposed to 19 °C and elevated nutrient concentration, growth rate was significantly higher (mean ± SD = 0.011 ± 0.003 gWW d^−1^, *N* = 5) compared to the mean growth rate for the rest of the treatments (mean ± SD = 0.007 ± 0.0019 gWW d^−1^, *N* = 15 Fig. 1).

**Table 1:** Summary of the analyses of variance for the growth rates: a) *Laminaria ochroleuca* and b) *Chondrus crispus* throughout the experiment

Source of variation		a) Growth of* Laminaria ochroleuca*			b) Growth of* Chondrus crispus*		
	df	MS	*F*	*p *value	MS	*F*	*p *value
Temperature (T)	1	**1.50 e**^**−5**^	**7.383**	0.015*	6.40 e^−8^	0.012	0.914
Nutrient (N)	1	2.23 e^−6^	1.095	0.310	**5.94 e**^**−5**^	**10.96**	0.004**
T x N	1	**1.16 e**^**−5**^	**5.698**	0.030*	**2.77 e**^**−5**^	**5.11**	0.038*
Residual	16	2.04 e^−6^			5.42 e^−6^		
Cochran C' test		*C* = 0.458 *p *value = 0.378			*C* = 0.480, *p *value = 0.306		
Transformation		None			None		
Test a posteriori		Temp x Nutrient			Temp x Nutrient		
		15C,N− > 19C,N−; all the other combinations no significant			19C,N + > 19C,N−; all the other combinations no significant		

Significant *p* values in bold

*p < 0.05, **p < 0.01, ***p < 0.001

### Fv/fm: maximum quantum efficiency

No significant differences were detected by the ANOVA in the case of *L. ochroleuca* (Table 2a). The maximum potential photosynthetic efficiency (Fv/Fm) in *C. crispus* showed a significant increase in the nutrient depletion treatment (Nut-) in comparison to the Nut + treatment, but no effects were found for temperature (Table 2b).

**Table 2:** Summary of the analyses of variance for the photosynthetic efficiency of a) *Laminaria ochroleuca* and b) *Chondrus crispus* throughout the experiment

Source of variation	df	a) Photosynthetic efficiency of *Laminaria ochroleuca*			b) Photosynthetic efficiency of* Chondrus crispus*		
		MS	*F*	*p *value	MS	*F*	*p *value
Temperature (T)	1	2.34 e^−5^	0.29	0.692	2.30 e^−4^	3.24	0.091
Nutrient (N)	1	3.75 e^−4^	2.75	0.127	**3.90 e**^**−4**^	**5.57**	0.031*
T x N	1	7.34 e^−5^	0.51	0.485	3.00 e^−5^	0.43	0.523
Residual	16	1.43 e^−4^			7.00 e^−5^		
Cochran C' test		*C* = 0.450 *p *value = 0.409			*C* = 0.486, *p *value = 0.286		
Transformation		None			None		
Test a posteriori		None			Nutrients		
					N− > N +		

Significant *p* values in bold

*p < 0.05, **p < 0.01, ***p < 0.001

### Primary productivity rates

#### Single component metabolic rates

#### Primary productivity

**Fig. 2:**
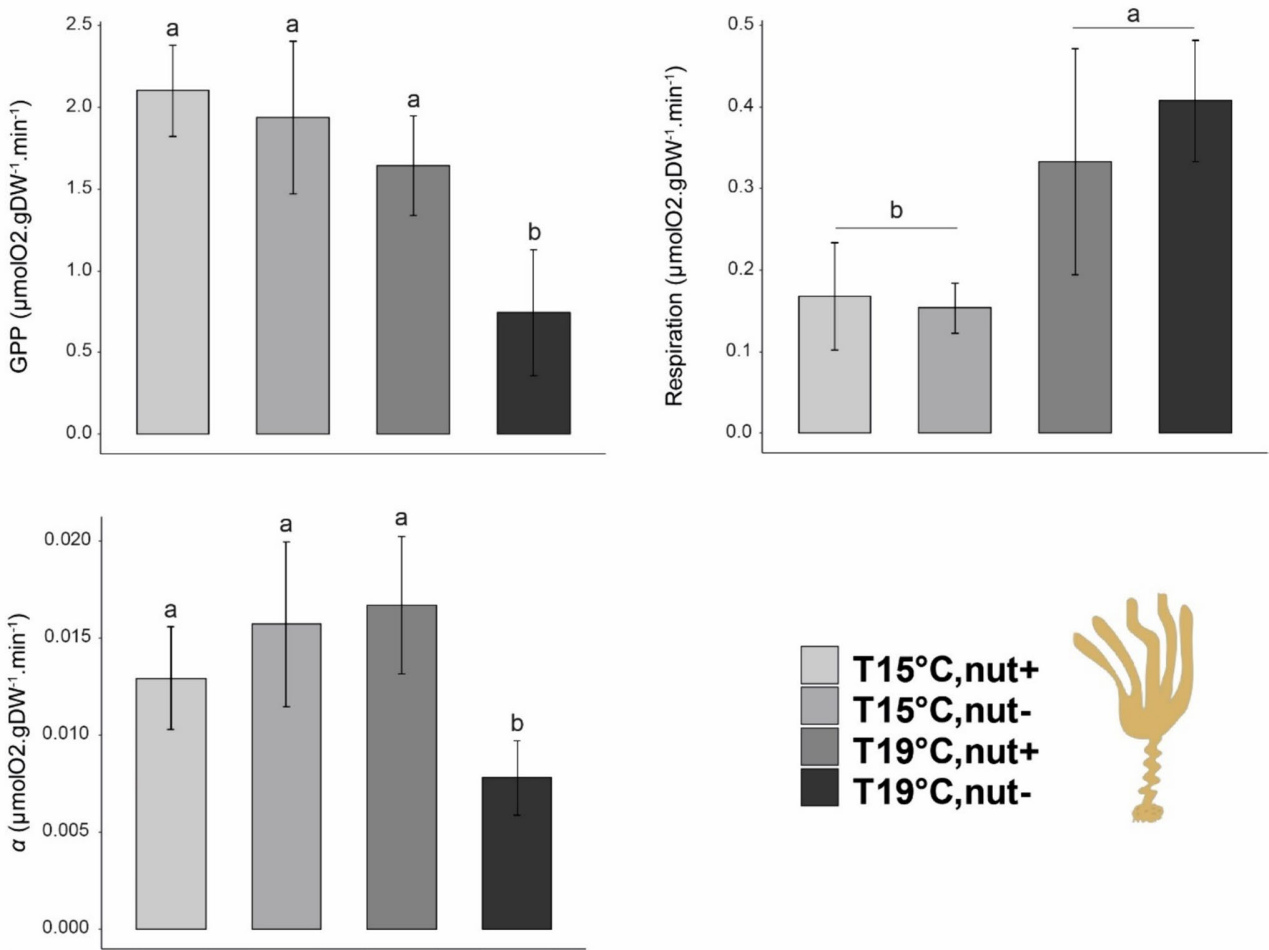
Mean of P–I curves parameters for *Laminaria ochroleuca* measured at the end of the experiment. Values are presented as means ± SD (*N* = 5). *p *values from two-way ANOVA (*p* ≤ 0.05). If interaction was significant, Tukey post hoc test was performed to detect differences between groups. Different letters denote for significant difference

**Fig. 3:**
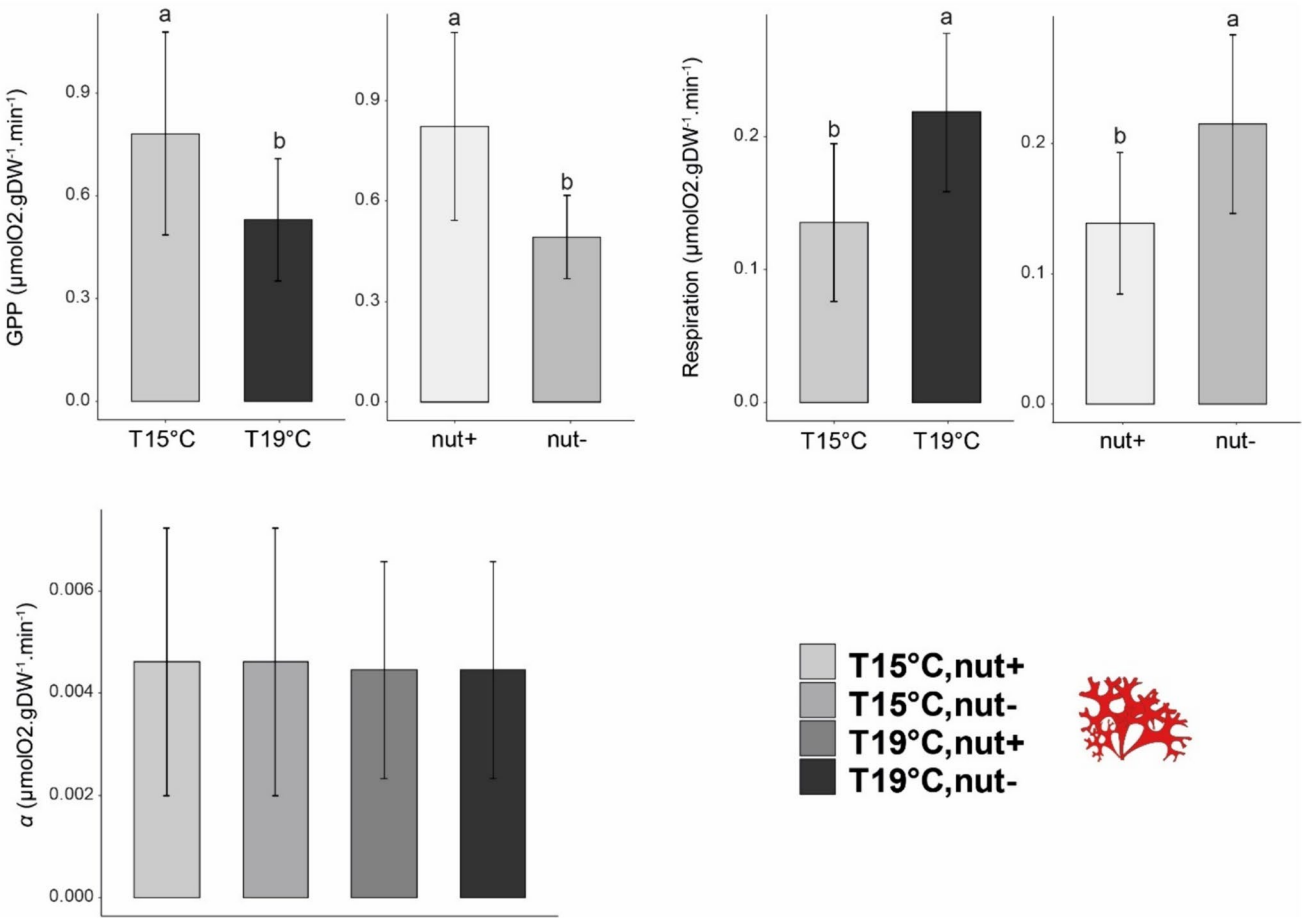
Mean of P–I curves parameters for *Chondrus crispus* measured at the end of the experiment. Values are presented as means ± SD (*N* = 5). *p *values from two-way ANOVA (*p* ≤ 0.05). If interaction was significant, Tukey post hoc test was performed to detect differences between groups. Different letters denote for significant difference

**Fig. 4:**
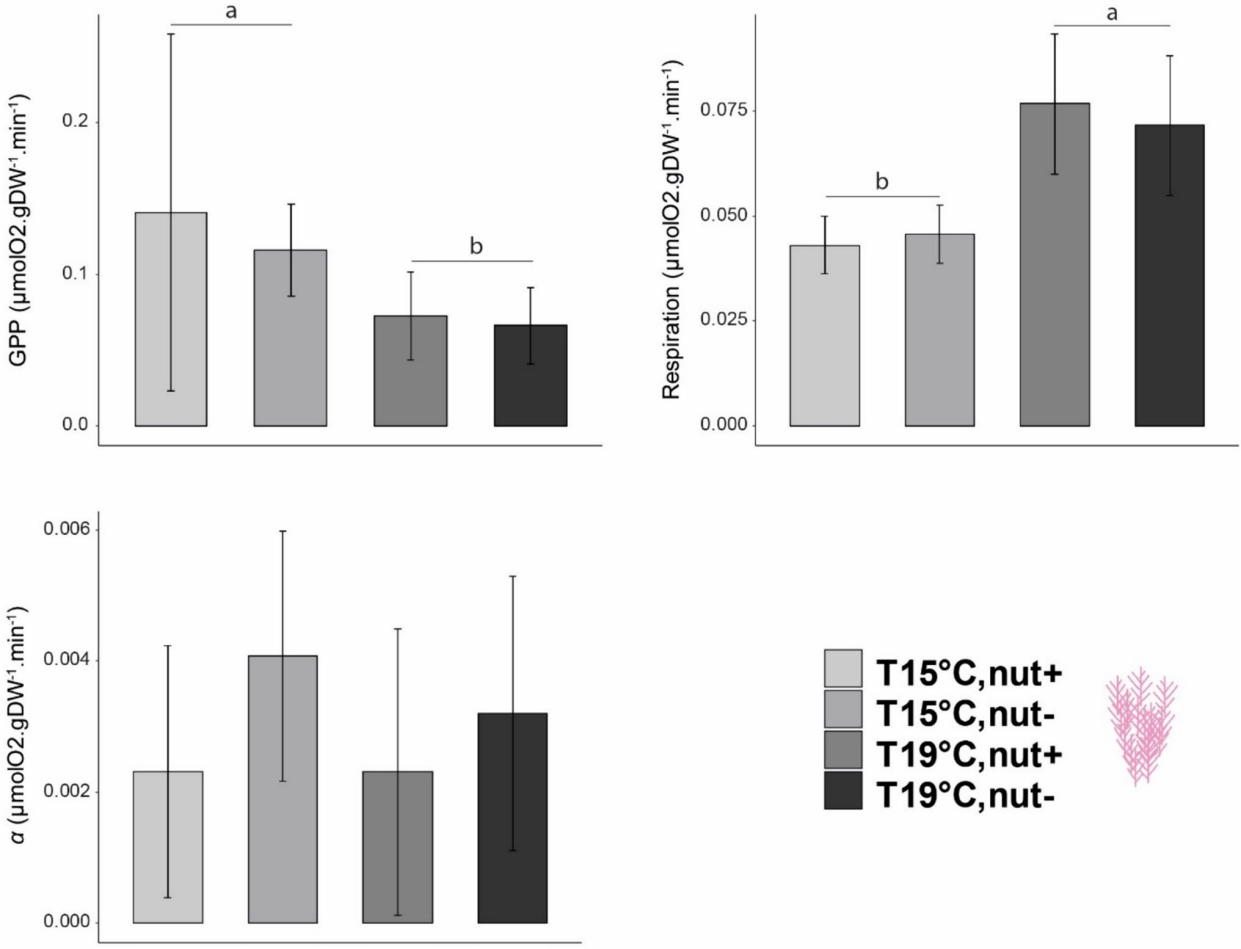
Mean of P–I curves parameters for the turf measured at the end of the experiment. Values are presented as means ± SD (*N* = 5). *p *values from two-way ANOVA (*p* ≤ 0.05). If interaction was significant, Tukey post hoc test was performed to detect differences between groups. Different letters denote for significant difference

Regarding maximum GPP (GPP_max_), values for *L. ochroleuca* ranged between 2.44 and 0.26 µmolO_2_ gDW^−1^ min^−1^ and were the highest among the components tested in our assemblages. Temperature and nutrient had a significant interactive effect on the kelp (i.e., *L. ochroleuca*) maximum productivity (Table 3a). Under high nutrient supply, no differences were detected between temperatures (overall mean ± SD = 1.87 ± 0.36 µmolO_2_ gDW^−1^ min^−1^, *N* = 5). However, in the nutrient depletion treatment, the temperature had a significant negative effect and at 19 °C, GPP values were lower than those measured at 15 °C (mean ± SD = 0.745 ± 0.38, and 1.937 ± 0.46 µmolO_2_ gDW^−1^ min^−1^, *N* = 5, respectively) representing a 60% decrease compared to 15 °C nutrient-enriched treatment (Fig. 2, Table 3a). *C. crispus* GPP was negatively affected by nutrient availability with lower values measured in the Nut- compared to the Nut + treatment (Fig. 3, Table 3b). Also, higher temperatures affected the thalli with GPP values were significantly higher for plants exposed to 15 °C compared to the one exposed to higher temperature (mean ± SD = 0.98 ± 0.36 and 0.65 ± 0.20 µmolO_2_ gDW^−1^ min^−1^, *N* = 10, respectively). Values of productivity for the turf significantly decreased with temperature with an average at 15 °C of 0.13 µmolO_2_ gDW^−1^ min^−1^ (± 0.08, *N* = 10) versus 0.07 µmolO_2_ gDW^−1^ min^−1^ (± 0.02, *N* = 10) at 19 °C (Fig. 4, Table 3c).

**Table 3:** Summary of the analyses of variance for the gross primary productivity of a) *Laminaria ochroleuca*, b) *Chondrus crispus*, c) turf and for d) the communities measured at the end of the experiment

Source of variation		a) *Laminaria ochroleuca*			b) *Chondrus crispus*			c) Turf			d) Communities		
	df	MS	*F*	*p *value	MS	*F*	*p *value	MS	*F*	*p *value	MS	*F*	*p *value
Temperature (T)	1	**3.49**	**25.33**	0.001***	**0.31**	**10.14**	0.005**	**6.0 e**^**−3**^	**7.39**	0.015**	0.52	2.00	0.176
Nutrient (N)	1	**1.40**	**10.46**	0.005**	**0.55**	**17.57**	0.001***	2.0 e^−4^	0.29	0.597	**2.27**	**8.70**	0.009**
T x N	1	**0.67**	**5.00**	0.040*	0.02	0.87	0.362	9.1 e^−4^	1.02	0.328	**1.25**	**4.80**	0.043*
Residual	16	**0.13**			0.03			8.9 e^−4^			0.261		
Cochran C' test		*C* = 0.404 *p *value = 0.6114			*C* = 0.686, *p *value = 0.019			*C* = 0.331, *p *value = 1.065			*C* = 0.386, *p *value = 0.707		
Transformation		None			None			None			None		
Test a posteriori		Temp x Nutrient			Temp & Nutrient			Temp			Temp x Nutrient		
		15C,N- > 19C,N−; 15C,N + > 19C,N;19C,N + > 19C,N−;all the other combinations no significant			N + > N−;15C > 19C			15C > 19C			19C,N + > 19C,N−;15C,N + > 19C,N−; all the other combinations no significant		

Significant *p* values in bold

*p < 0.05, **p < 0.01, ***p < 0.001

### Respiration rates

Respiration rate significantly increased with temperature for all the three components examined, but responses to nutrient concentrations varied by component. *L. ochroleuca* respiration rates increased by two-fold with a mean respiration rate of 0.161 µmolO_2_ gDW^−1^ min^−1^ (± 0.061, *N* = 10) when exposed to 15 °C and 0.344 µmolO_2_ gDW^−1^ min^−1^ (± 0.12, *N* = 10) at 19 °C (Fig. 2, Table 4a). *C. crispus* respiration rate was significantly higher at higher temperature but also higher in the Nut- treatment (Fig. 3, Table 4b). Respiration significantly increased when plants were exposed to 19 °C (mean ± SD = 0.219 ± 0.05 µmolO_2_ gDW^−1^ min^−1^, *N* = 10) compared to those exposed to 15 °C (mean ± SD = 0.135 ± 0.05 µmolO_2_ gDW^−1^ min^−1^, *N* = 10). For the nutrient treatment, respiration rates also increased in the nut- treatment (mean ± SD = 0.215 ± 0.07 µmolO_2_ gDW^−1^ min^−1^, *N* = 10) compare to nut + (mean ± SD = 0.139 ± 0.05 µmolO_2_ gDW^−1^ min^−1^, *N* = 10). Respiration rates for the turf were significantly higher at 19 °C (mean ± SD = 0.075 ± 0.015 µmolO_2_ gDW^−1^ min^−1^, *N* = 10) compared to the ones recorded at 15 °C (mean ± SD = 0.044 ± 0.006 µmolO_2_ gDW^−1^ min^−1^ *N* = 10) (Fig. 4, Table 4c).

**Table 4:** Summary of the analyses of variance for the respiration rates of a) *Laminaria ochroleuca*, b) *Chondrus crispus*, c) turf and for d) the communities at the end of the experiment

Source of variation		a) *Laminaria ochroleuca*			b) *Chondrus crispus*			c) Turf			d) Communities		
	df	MS	*F*	*p *value	MS	*F*	*p *value	MS	*F*	*p *value	MS	*F*	*p *value
Temperature (T)	1	**0.851**	**31.6**	**3.8 e**^−5^***	**0.03**	**15.93**	0.001**	**0.004**	**27.2**	**8.5 e**^−5^***	**0.02**	**4.86**	0.042*
Nutrient (N)	1	0.016	0.6	4.5 e^−1^	**0.03**	**13.31**	0.002**	8.07 e^−6^	0.05	0.826	4.0 e^−3^	0.96	0.341
T x N	1	0.031	1.2	3.0 e^−1^	1.0 e^−4^	0.08	0.777	7.55 e^−5^	0.5	0.506	3.55 e^−4^	0.09	0.773
Residual	16	0.027			2.0 e^−3^			1.63 e^−4^			0.004		
Cochran C' test		*C* = 0.637, *p *value = 0.043			*C* = 0.557, *p *value = 0.131			*C* = 0.428, *p *value = 0.500			*C* = 0.386, *p *value = 0.707		
Transformation		Square-root			None			None			None		
Test a posteriori		Temp			Temp & Nutrient			Temp			Temp		
		19C > 15C			N + > N−;15C > 19C			19C > 15C			19C > 15C		

Significant *p* values in bold

*p < 0.05, **p < 0.01, ***p < 0.001

### α-Alpha

When assessing the ability of the different components to use light under limiting conditions, individuals of *L. ochroleuca* showed a significant decrease in *α* when exposed to low nutrient concentration and elevated temperature. No significant effects were detected in the case of the turf and of *C. crispus* for the parameter alpha although the interaction was just marginally not significant and observed some trends toward significance with a decrease in the values in nut- when plants were exposed to high temperature (Table 5b and 5c).

**Table 5:** Summary of the analyses of variance for the α of a) *Laminaria ochroleuca*, b) *Chondrus crispus*, c) turf and for d) the communities throughout the experiment

Source of variation		a) *Laminaria ochroleuca*			b) *Chondrus crispus*			c) Turf			d) Communities		
	df	MS	*F*	*p *value	MS	*F*	*p *value	MS	*F*	*p *value	MS	*F*	*p *value
Temperature (T)	1	2.11 e^−5^	2.05	0.170	1.34 e^−7^	0.02	0.872	9.74 e^−7^	0.23	0.633	0.002	0.35	0.564
Nutrient (N)	1	**4.70 e**^**−5**^	**4.57**	0.048*	2.19 e^−6^	0.43	0.517	8.82 e^−6^	2.14	0.517	0.014	3.04	0.162
T x N	1	**1.70 e**^**−4**^	**16.62**	0.001***	1.97 e^−5^	3.94	0.064	9.22e^−7^	0.22	0.064	0.003	0.66	0.642
Residual	16	1.02 e^−5^			4.99 e^−5^			4.12 e^−6^			0.004		
Cochran C' test		*C* = 0.437, *p *value = 0.460			*C* = 0.476, *p *value = 0.317			*C* = 0.428, *p *value = 0.500			*C* = 0.395, *p *value = 0.655		
Transformation		None			None			None			None		
Test a posteriori		Temp x Nutrient			None			None			None		
		15C,N- > 19C,N−;19C,N + > 19C,N−; all the other combinations no significant											

Significant *p* values in bold

*p < 0.05, **p < 0.01, ***p < 0.001

### Whole community metabolic rates

#### Primary productivity

**Fig. 5:**
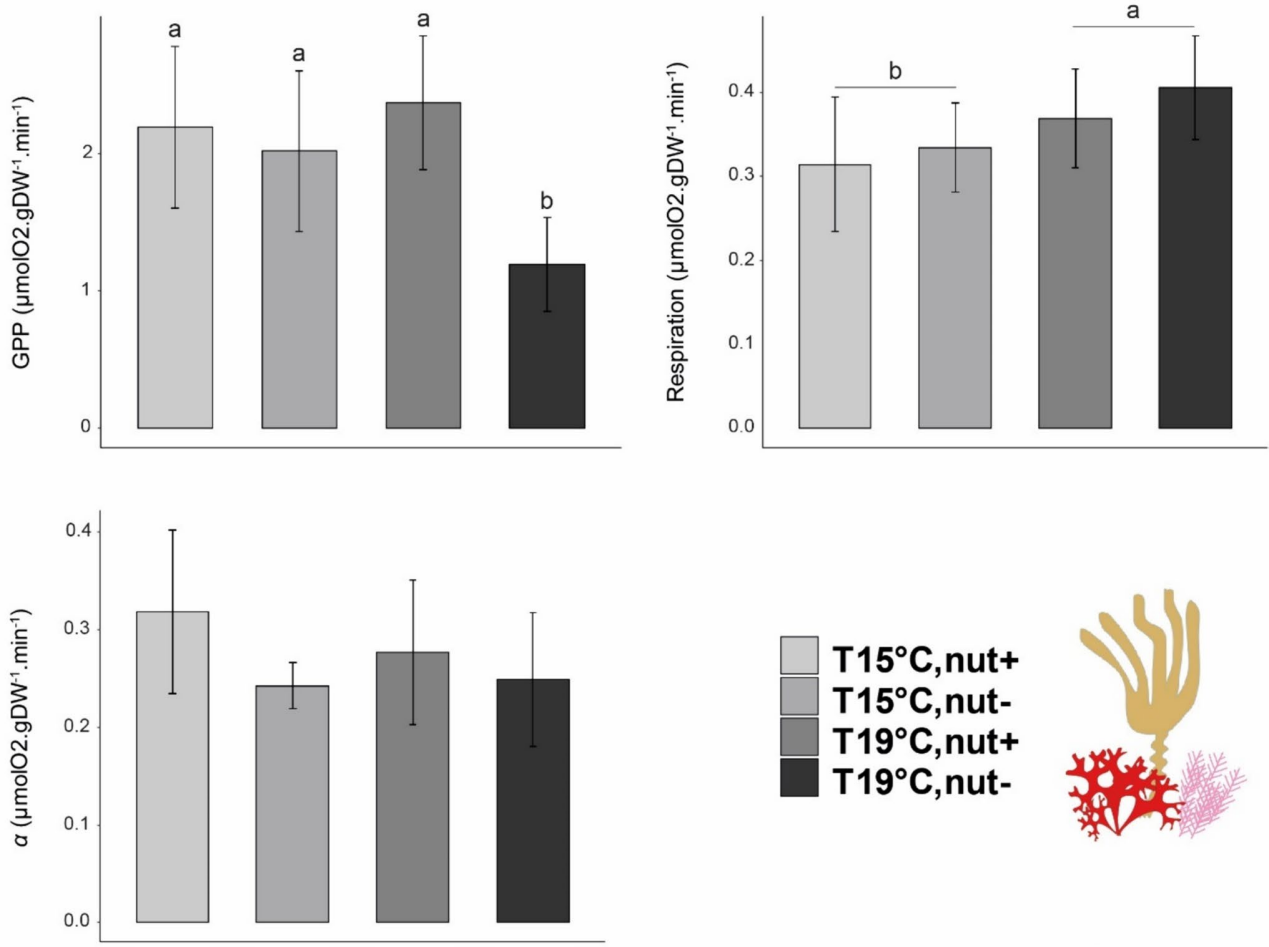
Mean of P–I curves parameters for the whole assemblage measured at the end of the experiment. Values are presented as means ± SD (*N* = 5). *p *values from two-way ANOVA (*p* ≤ 0.05). If interaction was significant, Tukey post hoc test was performed to detect differences between groups. Different letters denote for significant difference

Maximum GPP of the communities decreased significantly in the nut- treatment when exposed to 19 °C compare to the rest of the treatments, indicating a significant interaction between temperature and nutrients (Fig. 5, Table 3d). Within the Nut + treatment, values of P_max_ ranged between 1.04 and 3.15 µmolO_2_ gDW^−1^ min^−1^ (mean ± SD = 2.10 ± 0.56, *N* = 10) and no significant effect was detected between temperature. However, productivity significantly decreased in the Nut- treatment when communities were exposed to elevated temperature (mean ± SD = 1.19 ± 0.34, *N* = 5) compared to 15 °C (mean ± SD = 2.01 ± 0.54 µmolO_2_ gDW^−1^ min^−1^, *N* = 5) (Fig. 5, Table 3d).

### Respiration rate

The communities experienced significantly higher respiration rates when exposed to elevated temperature (Table 5d). Respiration of seaweeds exposed to 15 °C was in average 0.324 µmolO_2_ gDW^−1^ min^−1^ (± 0.06, *N* = 10), whereas seaweeds exposed to 19 °C displayed average respiration rates of 0.387 µmolO_2_ gDW^−1^ min^−1^ (± 0.06, *N* = 10). No other significant effects were detected (Fig. 5, Table 4d).

### α-Alpha

Values for α ranged between 0.16 and 0.43 (mean ± SD = 0.27 ± 0.06). No significant effects of treatment were detected on α (Fig. 5, Tables 5d).

## Discussion

Our experiment aimed to investigate how a less-intense coastal upwelling would affect coastal seaweed assemblages. We manipulated two of the foremost physical drivers projected to undergo alteration in response to a weakening upwelling scenario: temperature and nutrients. Experimental treatments were intentionally simple and did not pursue to replicate precisely future physical scenarios, which remain unclear regarding changes in the upwelling tendencies (Miranda et al. 2013; Sousa et al. 2020). Instead, we aimed to unravel trends and potential interactions among drivers. Our findings revealed that when confronted with weakened upwelling conditions (characterized by elevated temperatures and diminished nutrient availability), complex interactions among environmental factors could impact structure and functioning of coastal macroalgal communities. Furthermore, because of the high productivity of the kelp *L. ochroleuca*, most of the community-level responses were driven by the functional dominance of this species. Declines in large foundational species like *L. ochroleuca* may have shattering effects in coastal macroalgal productivity levels.

Growth is a useful response to assess the deleterious effects of environmental stressors because it integrates many biochemical and physiological effects and ultimately is linked to individual fitness (Piñeiro-Corbeira et al. 2019). In the case of the kelp *L. ochroleuca*, we found that when nutrients were available, temperature treatments did not affect the growth rate of this species. Indeed, *L. ochroleuca* is considered a warm-temperate Lusitanian species (Smale et al. 2014) and known as one of the most heat-tolerant species of the genus. In fact, the species has its southernmost limit of distribution in Morocco, where the water temperature can reach more than 20 °C (Izquierdo et al. 2002; Bartsch et al. 2008; Benazzouz et al. 2014). Previous studies have shown that *L. ochroleuca* is able to display optimal growth rates when exposed in the long term to temperature as high as 19 °C (Franco et al. 2018). Moreover, the ecotype from North Portugal exhibits positive growth rates after the short-term exposure to 27 °C, demonstrating its ability to withstand supra-optimal temperature (Pereira et al. 2015). Finally, thalli used in this study were collected in the rock pools from the low intertidal zone, where they are subject to a high range of physical stress and as result prone to withstand sub-optimal conditions than the individuals from the sub-tidal (Biskup et al. 2014). In an outdoor experiment using *L. ochroleuca* subjected to a thermal gradient and two nutrient levels, Franco et al. (2018) found additive, not interactive effects of these two drivers, with a significant reduction in the growth rate under nutrient limitation across the optimal temperature range. In our experiment, the effect of the drivers was not additive and growth rate was reduced to less than half in the Nut- e 19 °C treatment. Experimental conditions and/or the origin of the individuals may explain these differences, the experiment of Franco and colleagues ran under natural irradiance levels and with a large thermal gradient. Our indoor experiment had low irradiance levels and only two temperature levels. For example, there is evidence that nutrient limitation reduces capacity of seaweeds to screen out UV radiation, impairing the mechanism of energy dissipation and damage repair (Figueroa and Korbee 2010; Beardall et al. 2014), but in our indoor experiment, photo-damage was unlikely. The relevance of experimental irradiance level is stressed by the fact that Franco et al. (2018) recorded growth rates as high as 5 g FW/d, far surpassing those observed in the present study. Furthermore, the depth from which the thalli were collected could also play a role in these variations among studies. As emphasized by Biskup et al. (2014), intertidal thalli of *L. ochroleuca*, similar to the ones employed in our experiment, display significant differences in thermal stress tolerance when compared to the sub-tidal thalli used in Franco et al.'s (2018) study.

In the case of the sub-canopy species *C. crispus*, the interactive effect of temperature and nutrients was different than for *Laminaria*, with high-temperature treatment promoting growth only at the nutrient-enriched treatments. Growth might have been facilitated by high nutrient supply, allowing a faster metabolism to meet its demand (Davison 1991; Colvard and Helmuth 2017). *C. crispus* is a eurythermic species able to display optimal eco-physiological performance over a wide range of temperatures (Kübler and Davison 1993). In fact, optimal growth temperatures are described from 15 to 20 ºC and in the Iberian Peninsula even higher, up to 22 ºC (Piñeiro-Corbeira et al. 2019). Moreover, the species in Portugal is found in the low intertidal areas and rock pools, subjected to substantial variability in physical variables. This exposure to varying conditions could make it less vulnerable to temperature fluctuations (Kübler and Davison 1995). In the East North Atlantic, it can be found from the Norway to Portugal (Provan and Maggs 2011). However, a significant range contraction has been observed in the Iberian Peninsula in the last decades (Lima et al. 2007; Fernández 2016; Piñeiro-Corbeira et al. 2016). Whether this reduction in abundance is linked to changes in nutrient dynamics remains unknown. Some laboratory experiments suggest that nutrient limitation may constrain seaweed performance more than warming (Juanes and McLachlan 1992; Piñeiro-Corbeira et al. 2019). Interestingly, a recent detailed re-analysis of the changes in the seaweed communities’ composition published in Pineiro-Corberira (2016) identified nutrients as the main driver of temporal and spatial changes in those seaweeds communities (Vale et al. 2021).

Our observations regarding the growth of the two species measured were mostly in line with the results found for the metabolic rates. In particular, for *L. ochroleuca*, reduction of growth, productivity, and alpha indicated lower performances when both stressors were interacting. The reduction of productivity was significant with up to 60% minus in the 19 °C, Nut- compared to 15 °C, Nut + treatment. These results are consistent with previous studies, which have suggested a decrease in eco-physiological performance with decreasing nutrient concentration and increasing temperature in *Laminaria* species and other canopy-forming (Gerard 1997; Colvard and Helmuth 2017). In Franco et al. (2018), the reduction of growth associated with exposure to supra-optimal temperature was significantly lower under nutrient-enriched conditions than under nutrient-depleted situations, indicating a better resistance to high temperature when nutrients were available although those two factors did not show a significant interaction (see above). A similar dynamic was observed by Gao et al. (2013) for the kelp *Undaria pinnatifida*, which showed higher survival rate at higher temperatures when nutrients were abundant compared to when exposed to low nutrient concentrations. Additionally, Colvard & Helmuth (2017) have demonstrated that *Fucus vesiculosus* growing in nutrient-enriched water had higher P_max_ at high temperature than the ones lacking nutrients, suggesting that accessibility to nutrients increases thermal tolerance of this algae. In fact, nutrient limitation is recognized to negatively affect plants’ capacity to synthesize essential molecules such as chlorophyll, decreasing the PSII density and size (Gerard 1997), which ultimately impacts photosynthesis and growth (Wiencke and Bischof 2013). The decrease of performance when combined with high temperature might have resulted from increased metabolic rates, leading to higher nitrogen consumption, a requirement that cannot be met in a nutrient-deprived environment. Therefore, nutrient-limited plants will, faster, show signs of alteration of eco-physiological performances as they hardly meet their nutrient requirement to maintain the energetic demand of higher metabolic rate (Davison 1991; Colvard and Helmuth 2017). Furthermore, Gerard (1997) showed that the increase of protein content in non-nitrogen limited plants exposed to high temperatures could also be associated with the production of heat shock protein, promoting tolerance of high temperatures with increasing nutrient availability (Gerard 1997) and could partly explain the observed tolerance of *L. ochroleuca* to high temperature when exposed to sufficient nutrient availability.

In the case of the sub-canopy species, *C. crispus* and as it was stated above, the interactive effect of temperature and nutrients on growth showed high-temperature treatment promoting growth only at the nutrient-enriched treatments*.* However, in this species, the functional measures experimental drivers didn’t show any interaction among treatments. Respiration and productivity rates were affected by nutrient depletion and temperature in an additive way. The reduction in productivity associated was noticeably lower than for canopy species (around 30% at high temperature or nutrient-depleted treatments compared to optimal condition). Similarly, the turf was solely affected by temperature and no interactions were detected on the eco-physiological responses for this component. Temperature effects were negative with an increase in respiration and decrease in P_max_ rates.

As indicated above, community response followed a similar pattern as the canopy species response, with a similar significant decrease of productivity under low nutrient concentration and elevated temperatures. Only the effects of experimental treatments on alpha (i.e., photosynthetic efficiency at limiting irradiance levels) differed between *Laminaria* and the whole assemblage analyses. On the other hand, sub-canopy and turf seemed to be affected by the combination of the stressors to a smaller extent, and the low productivity associated with these components led to minor contribution to community response pattern. As expected from the metabolic rate-temperature relationship, the elevated temperature significantly increased the respiration rates for all the components of the assemblages and the whole assemblages themselves. As the temperature increased within the tolerance range of the species, the rate of enzyme-catalyzed reactions increased, resulting in a faster metabolism and ultimately higher respiration rates (Davison 1991). Primary productivity did not accompany this increase of respiration rates, and negative effects were exacerbated by nutrient depletion. Temperature effects were similar results to those observed by Tait and Schiel*.* (2013) in mesocosms, where naturally formed assemblage experienced increasing respiration rates with rising temperatures. Our results also show that the increase in respiration rate was not followed by increasing GPP like previously suggested in other marine autotrophs (Koch et al. 2013; Olabarria et al. 2013). However, this mismatch of respiration and GPP rates is a consequence from a reduced NPP when assemblages are exposed to higher temperature (Tait and Schiel 2013) and in our experiment when assemblages experienced high temperatures and low nutrient conditions. Overall, our results are consistent with the metabolic scaling theory and the relationship between metabolism and temperature in ectotherms and autotrophs, suggesting difficulty to sustain good functioning at larger scale (Brown et al. 2004; Kordas et al. 2011).

As foundation species, kelps have been reported to be the main contributor to community production, with a substantial reduction in the assemblages’ primary productivity when canopy species were removed (Schiel and Foster 2006; Davies et al. 2011). Additionally, responses of natural assemblages to experimental drivers have been shown to be modulated by the nature of the canopy-forming species, pinpointing the importance of canopy species contribution to assemblage response (Olabarria et al. 2013). In accordance with these previous studies, our results suggest that the overall effects of environmental changes on community productivity are expected to be highly dependent on the kelp fitness rather than other components with lower contribution to the productivity of the assemblage. Furthermore, sub-canopy species are highly dependent on the shade brought by the canopy (Flukes et al. 2014) and could be subject to photo-damage if this protection happens to disappear (Figueroa and Korbee 2010; Beardall et al. 2014), potentially aggravating the putative effects of weak upwelling conditions at the community level. The resilience to nutrient depletion of the turfs also suggests that species with poor ecological relevance could be given an advantage over canopy-forming species, potentially promoting shifts to turf-dominated assemblage (Filbee-Dexter and Wernberg 2018).

Surface temperature of the oceans has increased globally over the past decades. However, this increase is far from being homogenous and the North Atlantic seems to warm faster than other regions (Huang et al. 2017; Chan et al. 2019). In coastal areas influenced by eastern boundary upwelling systems (EBUS), those warming rates are partially buffered by cold upwelled deeper seawater (Seabra et al. 2019). Coastal ecosystems of North Portugal benefit from the NW Iberian upwelling system, which brings cold and nutrient-rich waters and reduces nearshore seawater warming (Mackas and Strub 2006). Extensive kelp forests thrive in these conditions, holding diversity rich and productive communities. However, the warming of the upper layer of the ocean is leading to an increase in the thermal stratification of the water column and a decrease in upwelling intensity (Sydeman et al. 2014; Sousa et al. 2020), lessening the efficiency of the upwelling in lifting nutrient-rich deep waters into the photic zone. The ecological effects stemming from shifts in the physical scenario could already be observable in the region. A study by De Azevedo et al. (2023) conducted in the same area as our research identified significant and rapid structural and functional transformations over the past decade. These changes were characterized by the increase in the abundance of warm temperature affinity species, particularly smaller seaweeds like turfs, in a process known as tropicalization. Our findings are consistent with these observed patterns and enable us to anticipate large functional effects derived from the loss of highly productive foundational kelp species. The primary productivity of coastal reefs dominated by seaweeds would experience a significant decline, leading to a cascading effect on higher trophic levels and the loss of fundamental ecosystem services (Smale et al. 2013).

Our understanding of the ecological impacts of changes in upwelling intensity is limited, rendering our capacity to foresee the future of these biologically rich and productive coastal ecosystems (García-Reyes et al. 2015). Despite its simplicity and the methodological limitations, our experiment provides valuable mechanistic clues into how communities will respond to changes in relevant upwelling-associated drivers, enabling the identification of the key contribution of foundational species like kelps to whole community responses (Tait et al. 2017). Casual understanding on the effect of drivers is fundamental to design effective management solutions to counteract the widespread trend of kelp forest degradation (Filbee-Dexter and Wernberg 2018).

### Supplementary Information

Below is the link to the electronic supplementary material.

Supplementary file1 (DOCX 17 KB)

## Data Availability

The datasets used and analyzed during the current study are available from reasonable request to Axel Chabrerie (axel.chabrerie@gmail.com).
