# Supplementary material for: What if the upwelling weakens? Effects of rising temperature and nutrient depletion on coastal assemblages
Source: Oecologia. 2024 Jun 5;205(2):365–81. doi: 10.1007/s00442-024-05571-6 (PMC11281971; doi:10.1007/s00442-024-05571-6)
Supplement: Supplementary file 1 — Supplementary file1 (DOCX 17 KB) [file 442_2024_5571_MOESM1_ESM.docx]

# **Electronic Supplementary Material**

# **What if the upwelling weakens? Effects of rising temperature and nutrient depletion on coastal assemblages.**

# Authors: Axel Chabrerie and Francisco Arenas

Benthic Ecology Team

Interdisciplinary of Marine and Environmental Research (CIIMAR)

University of Porto

**Supplementary Table 1**: Average dry weight (±Standard deviation) and percent of biomass of the assemblages used during the experiment.

| **Species** |  | | **Average dry weight g (SE)** | |  | |  | | **Average % biomass** | |
| --- | --- | --- | --- | --- | --- | --- | --- | --- | --- | --- |
| *Corallina* spp. |  | | 6.51 (3.17) | |  | |  | | 38.12 | |
| *Laminaria ochroleuca* |  | | 3.21 (1.31) | |  | |  | | 28.71 | |
| *Lithophyllum incrustans* |  | | 2.2975 (1.05) | |  | |  | | 20.53 | |
| *Chondrus crispus* |  | | 0.91 (0.34) | |  | |  | | 8.14 | |
| *Ceramium* spp. |  | | 0.58 (0.57) | |  | |  | | 5.25 | |
| *Chondracanthus acicularis* | | 0.47 (0.43) | |  | |  | | 4.22 | |  |
| *Ulva rigida* |  | | 0.36 (0.3) | |  | |  | | 3.20 | |
| *Chondracanthus tedeii* |  | | 0.24 (0.28) | |  | |  | | 2.20 | |
| *Ahnfeltiopsis devoniensis* |  | | 0.21 (0.25) | |  | |  | | 1.93 | |
| *Chondria dasyphylla* |  | | 0.16 (0.04) | |  | |  | | 1.46 | |
| *Sargassum muticum* |  | | 0.14 (0.05) | |  | |  | | 1.25 | |
| *Champia parvula* |  | | 0.11 (0.08) | |  | |  | | 0.98 | |
| *Dictyota dichotoma* |  | | 0.01 (0.009) | |  | |  | | 0.89 | |
| *Osmundea pinnatifida* |  | | 0.08 (0.04) | |  | |  | | 0.77 | |
| *Halopteris scoparia* |  | | 0.07 (0.06) | |  | |  | | 0.68 | |
| *Cryptopleura ramosa* |  | | 0.07 (0.04) | |  | |  | | 0.63 | |
| *Chondria coerulescens* |  | | 0.06 (0.01) | |  | |  | | 0.54 | |
| **Total biomass** |  | | 11.13 (3.33) | |  | |  | | 100.00 | |
